# Investigating nicotine pathway-related long non-coding RNAs in tobacco

**DOI:** 10.3389/fgene.2022.1102183

**Published:** 2023-01-19

**Authors:** Xiaodong Xie, Jingjing Jin, Chen Wang, Peng Lu, Zefeng Li, Jiemeng Tao, Peijian Cao, Yalong Xu

**Affiliations:** China Tobacco Gene Research Center, Zhengzhou Tobacco Research Institute of CNTC, Zhengzhou, China

**Keywords:** long non-coding RNA, nicotine biosynthesis, tobacco, co-expression, *Nicotiana tabacum*

## Abstract

Long non-coding RNAs (lncRNAs) are transcripts longer than 200 bp with low or no protein-coding ability, which play essential roles in various biological processes in plants. Tobacco is an ideal model plant for studying nicotine biosynthesis and metabolism, and there is little research on lncRNAs in this field. Therefore, how to take advantage of the mature tobacco system to profoundly investigate the lncRNAs involved in the nicotine pathway is intriguing. By exploiting 549 public RNA-Seq datasets of tobacco, 30,212 lncRNA candidates were identified, including 24,084 large intervening non-coding RNAs (lincRNAs), 5,778 natural antisense transcripts (NATs) and 350 intronic non-coding RNAs (incRNAs). Compared with protein-coding genes, lncRNAs have distinct properties in terms of exon number, sequence length, A/U content, and tissue-specific expression pattern. lincRNAs showed an asymmetric evolutionary pattern, with a higher proportion (68.71%) expressed from the *Nicotiana sylvestris* (S) subgenome. We predicted the potential cis/trans-regulatory effects on protein-coding genes. One hundred four lncRNAs were detected as precursors of 30 known microRNA (miRNA) family members, and 110 lncRNAs were expected to be the potential endogenous target mimics for 39 miRNAs. By combining the results of weighted gene co-expression network analysis with the differentially expressed gene analysis of topping RNA-seq data, we constructed a sub-network containing eight lncRNAs and 25 nicotine-related coding genes. We confirmed that the expression of seven lncRNAs could be affected by MeJA treatment and may be controlled by the transcription factor NtMYC2 using a quantitative PCR assay and gene editing. The results suggested that lncRNAs are involved in the nicotine pathway. Our findings further deepened the understanding of the features and functions of lncRNAs and provided new candidates for regulating nicotine biosynthesis in tobacco.

## Introduction

Long non-coding RNAs (lncRNAs) are a class of transcripts with a length of more than 200 nucleotides and have low or no protein-coding ability (Rinn and Chang, 2012). LncRNAs widely transcribed in the genome can be divided into natural antisense transcript (NAT) (Zhang et al., 2012; Jin et al., 2021), intronic non-coding RNA (incRNA), and long intergenic non-coding RNA (lincRNA) by the relative position of a lncRNA and the related protein-coding gene (Guttman et al., 2009). LncRNAs are transcribed mainly by RNA polymerase II in plants, and few can be transcribed by RNA polymerase IV and V (Wang and Chekanova, 2017).

In plants, a considerable number of lncRNAs related to the stress response (Qin et al., 2017; Sun et al., 2017), flowering suppression process (Heo and Sung, 2011; Henriques et al., 2017), fruit development (Kang and Liu, 2015), and fibre development (Salih et al., 2019) have been detected. A growing number of studies have confirmed that lncRNAs can regulate embryonic development and cell fate decision in various ways, such as modulation of chromatin modification and post-transcription regulation (Heo et al., 2013).

The rapid development of high-throughput sequencing technology has significantly reduced sequencing costs in recent years. Significant RNA-Seq data submitted by different research groups have accumulated in the NCBI SRA database (Coordinators, 2013), which makes up an excellent resource for the genome-wide identification of new function elements, including lncRNAs (Sun and Chua, 2019). The landscape of lncRNAs has recently been explored in many plant species, including maize, rice, tomato, and so on (Liu et al., 2012; Shuai et al., 2014; Wang et al., 2015; Deng et al., 2018; Huang et al., 2018; Wang et al., 2018).

Nicotine is the predominant alkaloid in tobacco (*Nicotiana tabacum*) (Saitoh et al., 1985), functioning as one of nature’s most effective plant defence metabolites. It is produced in the roots and accumulated mainly in the leaves (Solt, 1957). Many coding genes essential for nicotine biosynthesis have been identified, e.g., putrescine N-methyltransferase (PMT) (Biastoff et al., 2009), quinolinate phosphoribosyl transferase (QPT) (Saunders and Bush, 1979; Sinclair et al., 2000; Khan et al., 2017), berberine bridge enzyme-like proteins (BBLs), A622 (Kajikawa et al., 2011) and so on. Furthermore, several microRNAs (miRNAs) have been predicted to regulate nicotine biosynthesis (Guo et al., 2012; Qi et al., 2012; Tang et al., 2012; Li et al., 2015). Yet, little is known about the role of lncRNA in nicotine biosynthesis. Fan et al. found a novel non-coding nta-eTMX27, which acts as an endogenous target mimic (eTM) of tobacco miRNA nta-miRX27 and further affects *QPT2* transcription and the nicotine content in tobacco (Li et al., 2015). Recently, Chen et al. (2019) predicted miRNAs and circular RNAs (circRNAs) that may be involved in nicotine biosynthesis based on a topping dataset in tobacco. These case studies suggest the role of some lncRNAs in controlling nicotine biosynthesis. However, their study did not explore whether lncRNAs may be involved in nicotine biosynthesis at a pathway level.

To provide a more comprehensive set of tobacco lncRNAs, we investigated 549 public RNA-Seq datasets across different tissues and developmental stages. In total, 30,212 lncRNA candidates located in 19118 loci were identified; among them, 17326 lncRNA loci were high-confidence. Consistent with other studies (Wang and Chekanova, 2017), when compared with protein-coding genes, lncRNAs of tobacco also have distinct properties and tissue-specific expression patterns. Twelve NATs were predicted to be involved in the nicotine pathway by their host gene annotations. By weighted gene co-expression network analysis (WGCNA) (Langfelder and Horvath, 2008) and further filtering by the topping RNA-seq dataset, we constructed a sub-network containing eight lncRNAs and 25 nicotine-related coding genes. We confirmed that seven lncRNAs could be affected by MeJA treatment and transcription factor NtMYC2 using a quantitative PCR assay and gene editing. The results suggested that they are involved in the nicotine pathway. Our study provides a rich resource for lncRNA research in tobacco and uncovers a new role of lncRNA in mediating nicotine biosynthesis or transport.

## Materials and methods

### Dataset used for lncRNA identification

We collected the RNA-seq data of tobacco from the Sequence Read Archive of NCBI (Coordinators, 2013). The data covers 56 different SRA studies and ten distinct tissues (leaf, root, flower, anther, shoot, stem, petal, capsule, pollen, and seed), including 549 RNA-seq samples. There is a total of ∼2.91 TB SRA data, with sequence read lengths ranging from 33 to 488 nucleotides (Supplementary Table S1).

### Identification of lncRNAs

**FIGURE 1:**
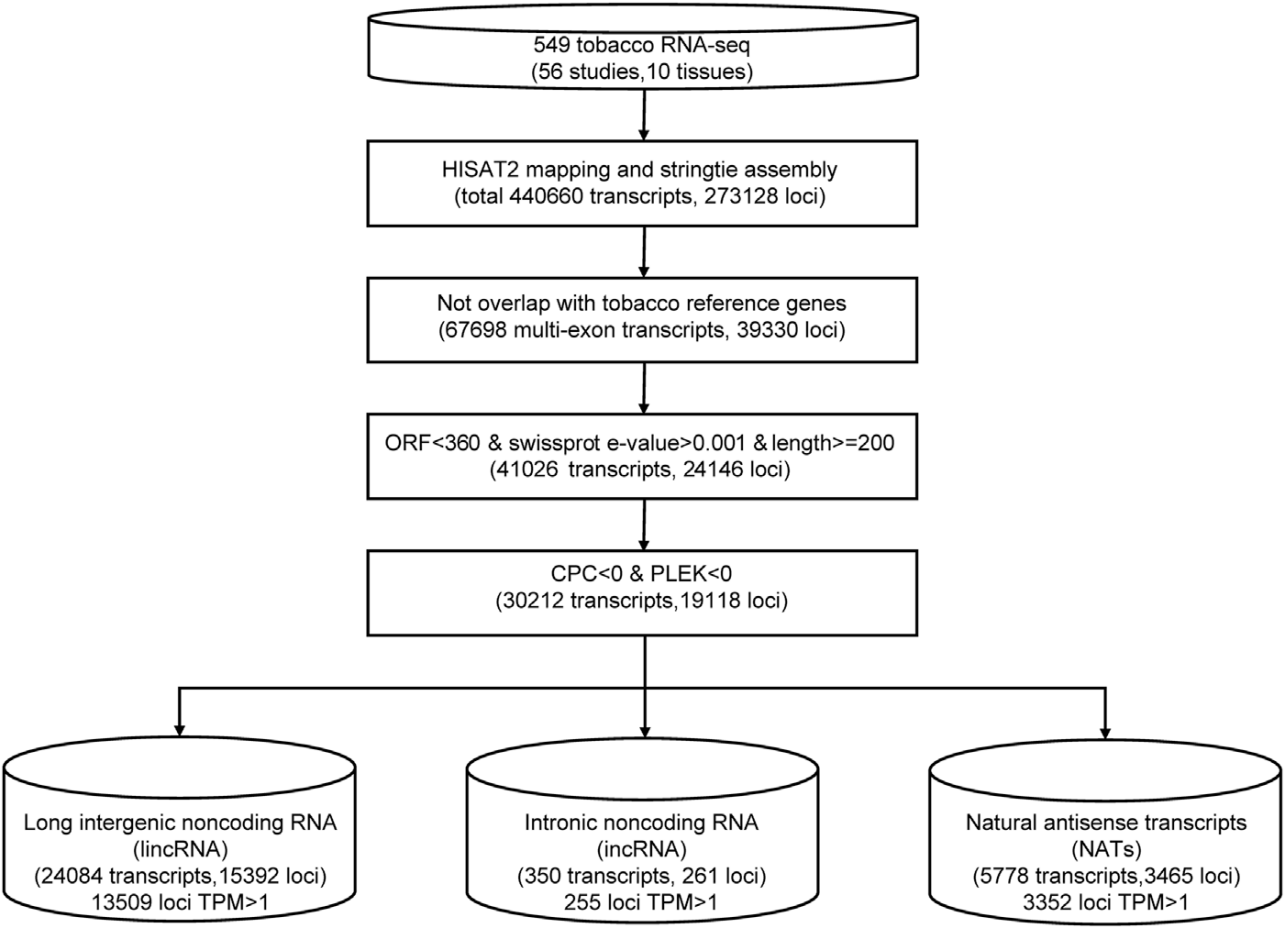
The pipeline for lncRNA identification in tobacco.

We merged transcript isoforms assembled from the different sequence datasets into one non-redundant set in tobacco; then, they were subjected to a series of filters to remove potential protein-coding genes (Figure 1). For the RNA-seq data, we trimmed all sequenced reads from each sample using the trim_galore program (Krueger, 2019) with a quality score of 30. Then, clean data were aligned to the tobacco reference genome (Edwards et al., 2017) using the read aligner HISAT2. The transcripts of each sample were assembled separately using stringtie, and all results of GTF files were merged into one with stringtie–merge (Pertea et al., 2015). Then, we compared the assembled transcript isoforms with the annotation of the tobacco reference genome (Sierro et al., 2014). Then, we discarded the transcripts with a length shorter than 200 bp and an open reading frame (ORF) longer than 120 aa. Swiss-Prot databases were searched using the blastx program (Altschul et al., 1990) to remove transcripts that may encode short proteins with the parameter -e 1.0e-4 -S 1. We calculated the remaining transcripts’ coding potential using the CPC (Kong et al., 2007) and PLEK (Li et al., 2014) programs. Only transcripts with both CPC and PLEK scores less than zero were used for the subsequent analysis. The remaining transcripts located in intergenic regions were identified as lincRNA candidates. If the transcripts were transcribed from the antisense strands of known genes, they were considered NAT candidates. The transcripts located in the intron region of known genes were identified as incRNA candidates.

### Sequence characters analysis of lncRNA and protein-coding gene

**FIGURE 2:**
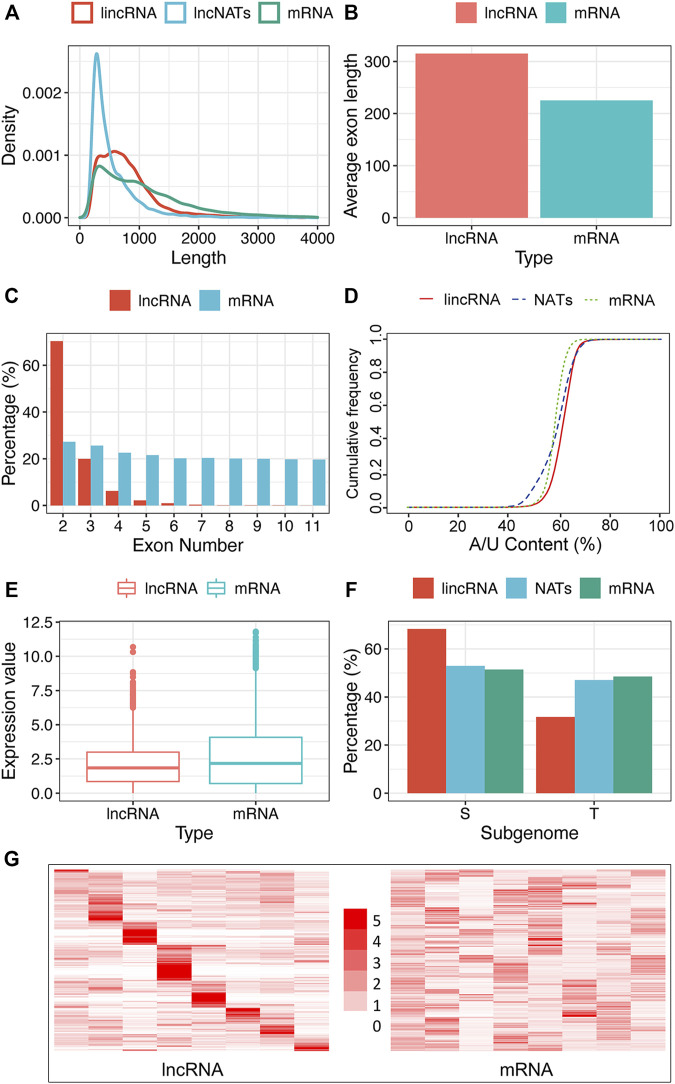
Characteristics of tobacco lncRNA. **(A)** Length distribution of coding genes (mRNAs), lincRNAs, and NATs, **(B)** Average exon length of lncRNA and mRNA, **(C)** Exon number of mRNAs, lincRNAs, and NATs, **(D)** A/U content of mRNAs, lincRNAs, and NATs, **(E)** Expression boxplot of mRNAs and lncRNAs, **(F)** Sub-genome dominance percentage of mRNAs, lincRNAs, and NATs between two ancestors, *Nicotiana sylvestris* (S) and *Nicotiana tomentosiformis* (T), **(G)** Tissue’s expression heatmap of lncRNAs and mRNAs clustered by row. The row represents TPM’s fold change relative to the sub-largest value in the row, and the column represents different tissue (From left to right are the tissues of the flower, root, dry capsule, anther, petal, leaf, stem and seed). Regarding the significant difference test, we used the *t*-Test method in figures b and e with a *p*-value of 0.05 and Pearson’s Chi-squared Test in figure f with a *p*-value of 1.0 e-5.

Sequence length vs. Density was counted and plotted in the R language (Figure 2A). We calculated the exon length of lncRNA and protein-coding gene by our Perl script and used the *t*-Test (*p*-value = 0.05) to test the significant difference (Figure 2B). The sequence proportion of lncRNA and coding-gene grouped by exon number was also computed and plotted in R language (Figure 2C). We counted the A/U proportion of lncRNA and protein-coding sequences by a Perl script and viewed it in the R language (Figure 2D). For comparing the expression distribution of lncRNAs and protein-coding genes, we performed 30 random selections with one sample at a time. The *t*-Test method was applied to the expression significant difference test (*p*-value = 0.05). Figure 2E shows the results of one randomly selected example.

### Expression analysis of lncRNA and protein-coding gene

To compare the expression patterns of lncRNA and protein-coding genes, we use transcripts per kilobase of exon model per million mapped reads (TPM) value to evaluate their expression level. Combined with the annotation of lncRNA and protein-coding genes, we calculated their TPM values in each sample using stringtie. If a gene is expressed more than twice as much in one of the tissues as all the others, which is defined as explicitly expressed. To compare tissue specificity, we calculated the expression matrices of protein-coding genes and lncRNAs, with samples in columns and genes in rows. We computed the fold changes of each expression value relative to the second-largest value by row. A Heatmap of the expression fold changes was plotted clustered by row in the R language (Figure 2G).

### Subgenome dominance analysis of lncRNA and protein-coding gene

The ancestors of common tobacco are *N.sylvestris* and *N.tomentosiformis*. To verify the transcripts expressed from which subgenome, the genome sequences of the two ancestors were downloaded from the Solanaceae Genomics Network FTP site (ftp.solgenomics.net/genomes) and merged into one file. We built a local blast database using the combined genome sequences. The sequences of lncRNAs and protein-coding genes in common tobacco were extracted and aligned with the previously made blast database with the parameter “-p blastn -v 1 -v 1 -K 1.” Then, the genome dominance of the lncRNAs and protein-coding genes were determined by species information of the blast best hit. We used Pearson’s chi-square test to test the significance of evolutionary asymmetry with a *p*-value of 1.0e-5. In our candidates, a few lncRNA transcripts belonged to a different lncRNA class but may have shared the same genomic locus; for example, there were 221 loci overlaps between lincRNA and NATs. The transcripts shared the same locus but were classified into different types; these were excluded when we computed the sub-genome dominance proportion.

### miRNA precursors’ prediction for lncRNAs

We downloaded one hundred sixty-four mature tobacco miRNAs from the miRBase database (Release 22) (Griffiths-Jones, 2006), and 376 novel miRNAs from previous studies (Chen et al., 2017) were collected. Mature miRNA sequences were aligned with all lncRNA using the blastn program (e-value = 1e-5). All subject sequences of the blast result were searched against the Rfam13.0 database (Kalvari et al., 2018) using cmscan (Nawrocki and Eddy, 2013) with the parameter -E 1.0e-3.

### miRNA mimics target prediction for lncRNAs

We predicted the miRNA mimic target using psRobot_mim software (Wu et al., 2013). Data from Wu et al. giving the sequences for A.thaliana and rice eTMs were downloaded (Wu et al., 2013). The miRNA pairing-site sequences of the predicted eTMs for each miRNA were extracted and aligned. MUSCLE (Edgar, 2004) and SeqLogo (Schneider and Stephens, 1990) were employed to generate multiple alignments and sequence logos of 35 predicted eTMs of Arabidopsis, rice, and tobacco for miR156.

### Prediction of lncRNA in cis or trans regulation

For incRNA, the target will be the host gene (Wu et al., 2018). The target for NATs will be the protein-coding gene in the opposite strand (Lapidot and Pilpel, 2006). lincRNA acted in a cis manner, as was predicted by determining whether the centre distance between neighbouring chromosomal genes and the lincRNA was less than 100 kb. The trans-acting lincRNAs and their protein-coding genes were predicted by calculating the correlation coefficient of expression with a 0.9 cutoff, and the lincRNAs that acted in a cis manner were excluded.

### Expression and co-expression analysis of lncRNAs and protein-coding genes

To get a relatively confident result, we selected some typical samples to conduct tissue-specific analysis and WGCNA. The main principles were “the same sequencing platform” and “more tissues were included in one project”. Differentially expressed lncRNAs and protein-coding genes between different tissues were analyzed using the one-way ANOVA method with a *p*-value less than 0.001. The regulatory relationship between differentially expressed lincRNA and protein-coding genes was subsequently investigated by weighted gene co-expression network analysis (WGCNA) using the SRP029183 data. We performed WGCNA using 2722 differentially expressed lncRNAs and 26707 protein-coding genes. First, we picked up the best threshold using the pickSoftThreshold function. We used the blockwiseModules function based on the above data with the parameters power = 9, minModuleSize = 30, mergeCutHeight = 0.25, and an unsigned scale-free topological network was constructed. To understand the functional roles of targets of lncRNAs, gene ontology (GO) (Ashburner et al., 2000) and Kyoto encyclopedia of genes and genomes (KEGG) (Kanehisa et al., 2021) pathway enrichment analysis was performed using the statistical methods of the hypergeometric distribution. GO terms and KEGG pathways with a corrected *p*-value less than 0.05 were significantly enriched.

### Gene network construction and visualization

Cytoscape (Shannon et al., 2003) was used to visualize the final interaction network.

### Tobacco treatment with methyl jasmonate

Tobacco seeds (K326) were sown on MS medium and grown with a 16/8 h light/dark photoperiod and 60% relative humidity at 28/25∘C. Then, 21-day-old seedlings grown on plates were exposed for 5 h to 100 μM MeJA. Seedlings were treated with 1% (v/v) Dimethyl sulfoxide (DMSO) without MeJA as a control. Root tissue was collected for RNA extraction, and qRT-PCR was conducted for eight lncRNA candidates involved in the nicotine pathway listed in Table 3.

### Creatation of NtMYC2 mutant line

For targeted NtMYC2 mutation, one sgRNA sequence of 20 nucleotides was designed (Supplementary Table S14). We used enzyme BsaI to digest plasmid pORE-Cas9, and the sgRNA was subcloned into the pORE-Cas9 vector and subsequently transformed into Agrobacterium tumefaciens (LBA4404) using the leaf disc method. DNA was extracted from T0 and T1 transgenic lines to detect mutations using DNeasy Plant Mini Kits (Qiagen, Hilden, Germany). The specific PCR primers are listed in Supplementary Table S14, and we performed mutant detection based on the methods in the previous literature (Xie et al., 2017).

### Validation of lncRNAs by qRT-PCR

To validate the lncRNA candidates we identified, we conducted quantitative real-time PCR (qRT-PCR) of 16 lncRNA candidates in four tissues (root, leave, stem and flower) of tobacco. RNA was isolated using a plant RNA rapid extraction kit (Genepure Plus, Imagene, China). The sample was converted to cDNA through RT-PCR with a reverse transcription kit (Takara Bio Inc., Shiga, Japan). The qPCR primers were designed by Primer 3.0. qRT-PCR was conducted in a total reaction volume of 15 μL, including 1 μL of specific primers (10 μM), 7.5 μL of the SYBR Green qPCR mix, 2 μL of the template cDNA (50 ng/μL), and 4.5 μL of dH2O. The reactants were mixed before the PCR. The GAPDH gene was used as the internal control, and qPCR analysis was performed using a CFX96TM Real-Time PCR Detection System (Bio-Rad, Hercules, CA, United States) following the manufacturer’s recommendations. The relative gene expression levels were evaluated using the 2−^△△ Ct^ method. The above techniques were also used to analyze the expression levels of all genes in the paper, and the primers used are listed in Supplementary Table S14. We performed a significant difference analysis of PCR results using the SNK.test function of the agricolae packet in R language, and the significance level was set to 0.05.

## Results

### Genome-wide discovery of 30212 lncRNA candidates in *N.tabacum*

We collected 549 RNA-seq datasets from public resources across ten distinct tissues (leaf, root, flower, anther, shoot, stem, petal, capsule, pollen, and seed) (Supplementary Table S1). To characterize tobacco lncRNAs, we developed a computational identification pipeline based on whole transcriptome data (Figure 1). After mapping to a reference genome, the tobacco transcripts were reconstructed from all RNA-seq datasets using stringtie (Pertea et al., 2015). We got a total of ∼440,000 transcripts. We applied four filter processes to distinguish lncRNAs from protein-coding transcript units. First, we removed transcripts that overlapped with known protein-coding genes (84.45%). Second, we filtered transcripts with a length of <200 nucleotides (nt). Then, we removed transcripts with high sequence similarity to known proteins or with an open reading frame (ORF) length of more than 360 nt. Last, we used the Coding Potential Calculator (CPC) (Kong et al., 2007) and PLEK (Li et al., 2014) to predict the coding potential of leftover transcripts and discarded transcripts with a score of more than zero. Finally, 30,212 lncRNA candidates were identified (Supplementary Table S2). Based on their genomic location relative to protein-coding genes, these lncRNAs were further classified into 24,084 (79.72%) lincRNAs, 5778 (19.12%) antisense lncRNAs, and 350 intronic lncRNAs (1.16%) (Figure 1; Supplementary Table S2). Among them, 17326 lncRNA loci with a TPM value greater than one at least in one sample were defined as high-confidence lncRNA loci.

### LncRNAs have distinct properties compared with protein-coding genes

To more clearly characterize tobacco lncRNAs, we compared lincRNAs and NATs with protein-coding genes. As shown in Figure 2A, the overall length of the lncRNAs was shorter than that of mRNAs. The exon number of the lncRNAs was less than that of the mRNAs (Figure 2C), which is similar to findings in other plants (Li et al., 2019; Tian et al., 2019), but the average length of the exons in the lncRNAs was substantially longer than that of mRNAs (Figure 2B). As for exon numbers, approximately 88% of the lincRNAs and 77% of the NATs contained less than three exons, whereas only 50% of the protein-coding genes had less than three exons (Figure 2C). Interestingly, the tobacco lncRNAs showed more A/U-rich regions relative to the protein-coding gene (Figure 2D), and most lncRNAs had relatively lower expression than protein-coding genes (Figure 2E). We performed subgenome dominance analysis of the lncRNAs in allotetraploid tobacco by comparing the genomic sequence similarity with its two ancestral species *Nicotiana sylvestris* and *Nicotiana tomentosiformis*. We found that most lincRNA genes (10,548 loci, 68.71%) were expressed from the *N.sylvestris* (S) subgenome and 4803 lincRNA genes (32.29%) were from the *N.tomentosiformis* (T) subgenome. The proportions of NATs and protein-coding genes between the subgenomes were similar: 1706 (52.12%) NATs were from the S subgenome, and 1567 (47.87%) NATs were from the T sub-genome, whereas there were 33,742 (48.55%) and 35,758 (51.45%) protein-coding genes expressed from the T and S sub-genomes, respectively (Figure 2F). This observation indicated that lincRNA evolved asymmetrically, more significantly than the protein-coding gene.

### LncRNAs show highly tissue-specific expression pattern

We systematically estimated the expression levels of lncRNAs and protein-coding genes using the transcripts per kilobase million (TPM). The results showed that most lncRNAs had lower expression levels than protein-coding genes (Figure 2E). We explored the tissue-specificity of lncRNA in expression level using the RNA-seq data from eight tissues of 25 samples (Supplementary Table S3). We found that 15.08% of lncRNAs were detected in only one of the tissues (TPM>1), whereas 19.6% of lncRNAs expressed in five or more tissues. By contrast, only 6.28% of protein-coding genes were expressed in one tissue alone, and 60.36% of protein-coding genes were detected in five or more tissues using the same criteria (Figure 2G). The reproductive tissues (dry capsule, anther, and petal) had more tissue-specific lncRNAs than other tissues (Figure 2G). The tissue-specific expression pattern for lncRNAs suggests that the expression of these sequences is biologically controlled rather than simply reflecting “transcriptional noise.” RT-PCR results (Figure 6A) showed that the specific pattern was broadly consistent with the RNA-seq data. For example, lncRNA (MSTRG.173387) was detected as tissue-specifically expressed in roots by RNA-seq and RT-PCR (Figure 6A).

### LncRNAs as precursors and potential mimic targets of miRNAs

Recent studies indicated that lncRNA could act as a miRNA precursor (Wu et al., 2013). In our study, 104 lncRNA transcripts were detected as precursors of 30 known miRNA family members, including miR156, miR159, miR160, miR162, and miR164, whereas 68 lncRNA members were identified as precursors of 49 novel miRNA in tobacco (Supplementary Table S4).

**FIGURE 3:**
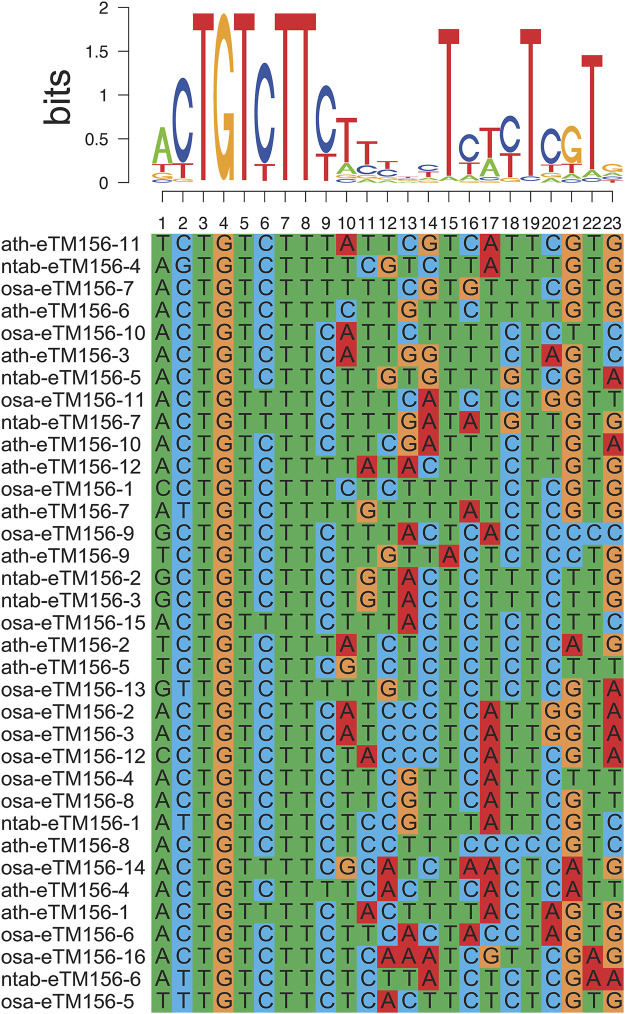
Alignment of lncRNAs as eTMs of miRNA156 in tobacco, rice, and Arabidopsis.

Studies have shown that lncRNA can act as an endogenous target mimic (eTM) to regulate miRNA functions by binding to miRNA *via* complementary sequences, blocking the interaction between miRNA and its authentic target (Wu et al., 2013). Such inhibition of miRNA activity is termed target mimicry. Similar to the interactions of miRNAs with their targets, miRNA target mimicry also relies on the sequence-dependent interaction of miRNA with lncRNA, except for the bugles in the middle of miRNA-lincRNA duplexes. In this research, 73 lncRNA genes (110 transcripts) were predicted to be the potential eTMs for 39 miRNAs (Supplementary Table S5), mostly 21 nucleotides (nt) in length. The eTMs of miR156 were recently screened in *Arabidopsis thaliana* (Wu et al., 2013). Sequences of the predicted eTM-binding sites for the same miRNAs in Arabidopsis and rice were aligned to confirm the predicted results. We found the eTM-binding sites for the same miRNAs to be well-conserved in tobacco and Arabidopsis/rice (Figure 3). Therefore, we proposed that specific interactions between these potential eTMs and miRNAs may exist and play a fundamental role in plants.

### The cis and trans roles of lncRNAs in target genes

**FIGURE 4:**
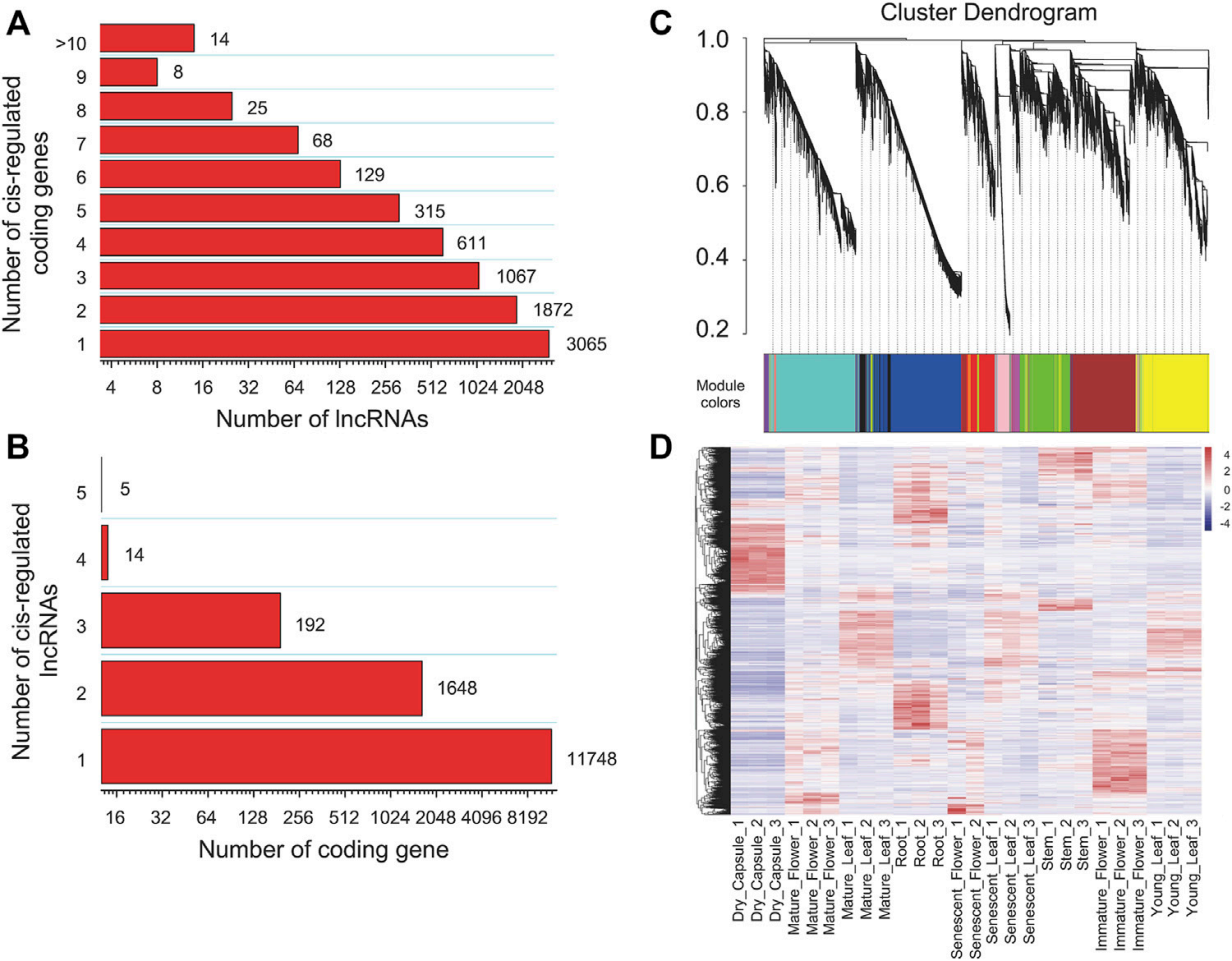
Interaction statistics of lncRNAs and their target protein-coding genes in tobacco. **(A)** The number of target protein-coding genes regulated by lncRNAs, **(B)** The number of lncRNAs that have potential trans-regulatory effects on protein-coding genes, **(C)** Clustering dendrograms of lncRNAs and their tans-regulated targets, with dissimilarity based on the topological overlap, together with assigned module colours, **(D)** Hierarchical cluster of expression for lncRNAs in different tissues.

To investigate the functions or biological processes of lncRNAs, we tried to predict their cis and trans targets. For the cis analysis of the lncRNAs, we searched coding genes 100 kb upstream and downstream of lincRNAs. The results indicated 7,165 lincRNAs with potential cis-regulatory effects on 13,607 protein-coding genes in 15,701 gene pairs. Among them, ten protein-coding genes targeting 13 lincRNAs were involved in the nicotine pathway (Supplementary Table S6). Among the cis network, 480 and 689 are located within 5 kb upstream and downstream of annotated genes. We found that 4,109 (57.35%) lincRNAs have more than one co-localized gene, of which 49.55% target two to four target genes and only 14 lincRNAs have more than eight target genes (Figure 4A). Up to 11,748 (86.33%), protein-coding genes corresponded to just one lncRNA, and only five protein-coding genes were cis-regulated by up to five lncRNAs (Figure 4B). In addition, we calculated the expression Pearson correlation between lincRNA and its cis-regulated target gene pair. When 0.9 was set as the correlation coefficient cutoff, the expression of 61 lincRNA genes and their corresponding 65 target genes were strongly correlated (Supplementary Table S7).

For the trans analysis of the lncRNAs, 4,947 lincRNAs with 27,649 associated target protein-coding genes were determined to be trans-regulated in 1,711,276 gene pairs (Supplementary Table S8), in which 1,711,167 pairs were positively correlated, and only 109 gene pairs were negatively correlated. Gene annotation indicated that 53 target-coding genes correspond to 567 lincRNAs known to be involved in nicotine biosynthesis or transport (Supplementary Table S9).

**FIGURE 5:**
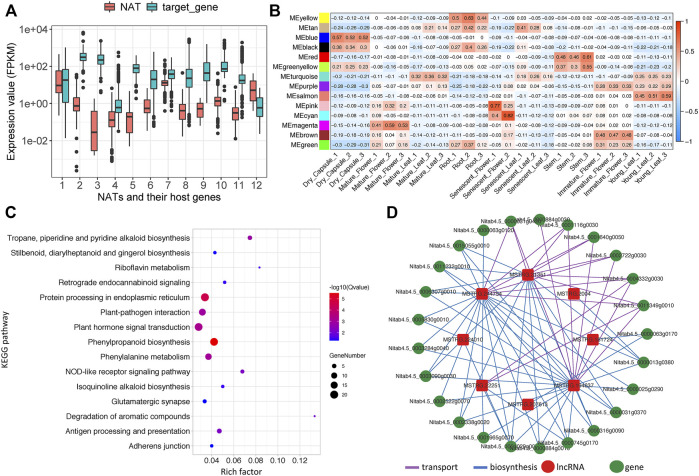
Analysis of lncRNAs associated protein-coding gene network related to nicotine pathway. **(A)** Expression boxplot of 12 NATs and their corresponding host genes, **(B)** Module-sample associations. Each row corresponds to a module eigengene, a column to a sample. Each cell contains the corresponding correlation, **(C)** KEGG enrichment plot of topping respond genes **(D)** The lncRNA-coding gene subnetwork related to the nicotine pathway.

We subsequently investigated the regulatory relationship between lincRNA and the protein-coding gene by weighted gene co-expression network analysis (WGCNA). In total, 2,771 lncRNAs and 26,705 protein-coding genes formed a complex network. The co-expression network was divided into 14 modules (Table 1; Figures 4C, 5B; Supplementary Table S10), which contained different proportions of lncRNAs, ranging from one in module MEcyan to 785 in MEblue, with an average of 7.14% (Table 1). Several distinguishable patterns were found for some modules (Figure 4D). For example, the MEyellow module contained 4,385 protein-coding genes and 649 lincRNAs, which showed high specificity in root tissue (Figure 5B).

**TABLE 1:** The statics of modules in WGCNA results.

Module name	Module color	LincRNA number (%)	Coding gene number (%)
MEturquoise	turquoise	522 (18.84%)	5366 (20.09%)
MEpink	pink	46 (1.66%)	859 (3.22%)
MEblue	blue	785 (28.33%)	4963 (18.58%)
MEgreen	green	210 (7.58%)	2921 (10.94%)
MEyellow	yellow	632 (22.81%)	4116 (15.41%)
MEred	red	208 (7.51%)	1557 (5.83%)
MEsalmon	salmon	5 (0.18%)	130 (0.49%)
MEblack	black	56 (2.02%)	867 (3.25%)
MEbrown	brown	217 (7.83%)	4581 (17.15%)
MEpurple	purple	11 (0.4%)	287 (1.07%)
MEmagenta	magenta	34 (1.23%)	545 (2.04%)
MEtan	tan	27 (0.97%)	181 (0.68%)
MEgreenyellow	greenyellow	17 (0.61%)	269 (1.01%)
MEcyan	cyan	1 (0.04%)	63 (0.24%)

### Identification of putative lncRNAs involved in the nicotine pathway

Commonly, incRNA will regulate its host gene (Wu et al., 2018), whereas the target for NATs will be the protein-coding gene in the opposite strand (Lapidot and Pilpel, 2006). Unfortunately, our study found no incRNAs for known nicotine biosynthesis genes. However, 12 NATs were identified that might regulate 11 known nicotine biosynthesis-related genes, while one NAT may target nicotine transport genes (Table 2). Among these NATs-target pairs, almost all the target genes were higher than their NATs, except for one pair (MSTRG.140254 and *SAMDC*, x = 12) (Figure 5A).

**TABLE 2:** The NATs are involved in nicotine biosynthesis or transportation.

Index	NAT ID	NAT host gene ID	Host gene name	Bioprocesses
1	MSTRG.2004	Nitab4.5_0000013g0380	PMT	nicotine biosynthesis
2	MSTRG.20132	Nitab4.5_0000259g0260	SAMDC	nicotine biosynthesis
3	MSTRG.45350	Nitab4.5_0000895g0150	SAMS	nicotine biosynthesis
4	MSTRG.49269	Nitab4.5_0001029g0080	BBL	nicotine biosynthesis
5	MSTRG.57034	Nitab4.5_0001317g0070	ADC	nicotine biosynthesis
6	MSTRG.68671	Nitab4.5_0001810g0050	NUP1	nicotine transportation
7	MSTRG.82612	Nitab4.5_0002539g0040	NtMYC	nicotine biosynthesis
8	MSTRG.94376	Nitab4.5_0003280g0010	ODC	nicotine biosynthesis
9	MSTRG.118406	Nitab4.5_0005266g0020	ADC	nicotine biosynthesis
10	MSTRG.129894	Nitab4.5_0006589g0020	SAMS	nicotine biosynthesis
11	MSTRG.139749	Nitab4.5_0008037g0020	QS	nicotine biosynthesis
12	MSTRG.140254	Nitab4.5_0008122g0010	SAMDC	nicotine biosynthesis

We performed co-expression analysis for lincRNAs and protein-coding genes by computing the Pearson correlation coefficient (PCC). With a 0.85 cutoff for PCC, 1,113 lincRNAs and 67 associated nicotine-related genes were determined (Supplementary Table S12). It is well-known that nicotine is a secondary metabolite exclusively synthesized in roots. We identified the MEyellow module is specific in root tissue. It contains many nicotine-related coding genes, and 29 well-known genes involved in nicotine biosynthesis or transport (for example, *PMT*, *MPO*, *A622*, *MATE*, *ERF189*, *AO*, *SAMS*, *BBLs*, and *QS*) were in this module (Supplementary Table S11). So, we proposed that some lincRNAs in this module may be involved in nicotine biosynthesis or transportation. After filtering the results of the PCC valued less than 0.85, 325 lincRNA-targeting 19 nicotine biosynthesis genes and 296 lincRNA-targeting 13 nicotine transport genes were identified (PCC>0.85&MEyellow = yes, 344 unique lincRNA in total) (Supplementary Table S12).

Nicotine biosynthesis can be induced by topping or leaf wounding in tobacco plants (Kutchan, 1995; Sinclair et al., 2004). Differentially expressed genes (DEGs), including coding and non-coding genes caused by topping, were analyzed using our in-house topping RNA-seq data. Only the loci with a max mean value greater than one in topping samples were considered, combined with the parameter of a *p*-value <0.05. We found 9,556 coding and non-coding DEGs, of which 717 DEGs belonged to the MEyellow module. 571 DEGs (34 lncRNAs and 537 coding genes) were upregulated (Supplementary Table S13), and only 146 DEGs (four lncRNAs and 142 coding genes) were downregulated. KEGG pathway enrichment analysis showed that “Tropane, piperidine and pyridine alkaloid biosynthesis,” “Plant hormone signal transduction,” “Isoquinoline alkaloid biosynthesis” pathways, and so on were significantly enriched (Figure 5C). GO enrichment analysis of the biological processes showed that nicotine biosynthetic process (GO:0042179), basipetal auxin transport (GO:0010540), regulation of signal transduction (GO:0009966), jasmonic acid-mediated signaling pathway (GO:0009868) and so on were significantly enriched (Supplementary Table S13).

In our 12 identified NATs and 344 lincRNA candidates, seven lincRNAs and one NAT (ID: MSTRG.2004)-targeting PMT (ID: Nitab4.5_0000013g0380) gene were significantly induced by topping (log2FDC>1 & p.adjust<0.05) (Table 3). We build a sub-network with nicotine pathway-related coding genes using these lncRNAs mentioned above. In this sub-network, MSTRG.244754, MSTRG.31351, and MSTRG.194637 have more links than other nodes (Figure 5D), indicating that these lncRNAs may play essential roles in nicotine biosynthesis.

**TABLE 3:** Potential lncRNAs involved in nicotine biosynthesis in MEyellow module and induced by topping.

Type	Gene ID	log2FDC (after/before)	*p*-value
lincRNA	MSTRG.181724	1.5239	0.0202
lincRNA	MSTRG.194637	1.3279	0.0026
lncNATs	MSTRG.2004	6.9582	0.0002
lincRNA	MSTRG.207613	1.0736	0.0242
lincRNA	MSTRG.22251	1.0946	0.0128
lincRNA	MSTRG.224010	1.0901	0.0243
lincRNA	MSTRG.244754	1.7496	0.0189
lincRNA	MSTRG.31351	1.0313	0.0059

### Nicotine-related lncRNA candidates were affected by MeJA treatment, and the transcription factor NtMYC2

**FIGURE 6:**
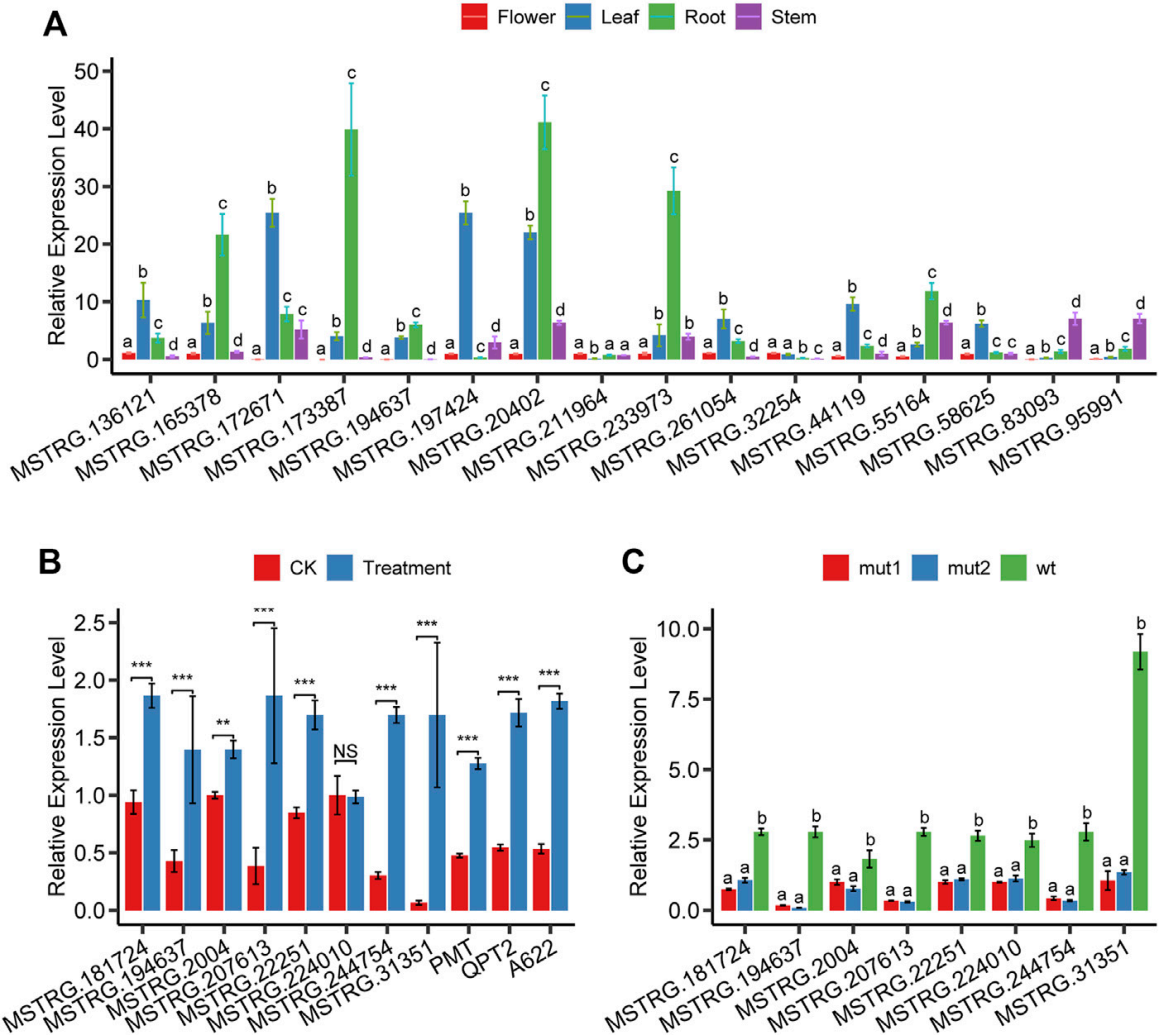
The relative expression level of lncRNAs and genes. **(A)** expression of 16 randomly selected lncRNAs in flower, leaf, root, and stem tissues, **(B)** expression of eight lncRNAs and three well-known nicotine biosynthesis protein-coding genes in root tissue after MeJA treatment, **(C)** expression of seven lncRNAs in root tissue of NtMYC2 mutation lines.

Topping and wounding induce nicotine biosynthesis through mediating phytohormones, mainly jasmonate (JA) and auxin (Baldwin et al., 1994). To confirm that the nicotine-related lncRNAs we found respond to JA, we analyzed the expression changes after MeJA treatment using qPCR. Results showed that 7 (MSTRG.181724, MSTRG.194637, MSTRG. 2004, MSTRG.207613, MSTRG.22251, MSTRG.244754, MSTRG.31351) of 8 lncRNA’s expression levels were significantly upregulated except MSTRG.224010 (Figure 6B). In addition, we performed expression analysis on three protein-coding genes (PMT, QPT2, A622) known to play an essential role in the nicotine biosynthesis pathway; as expected, the expression of all these three coding genes was significantly upregulated after MeJA treatment (Figure 6B).

Jasmonate-inducible nicotine formation in Nicotiana plants is suppressed by tobacco JAZ proteins (Shoji et al., 2008) and is regulated by both MYC2-related and NIC2-locus ethylene response factor (ERF) transcription factors. NtMYC2 controls nicotine biosynthesis genes in two combinatorial ways: directly binding the G-box in the target promoters and up-regulating the NIC2-locus ERF genes (De Boer et al., 2011; Shoji and Hashimoto, 2011). To confirm whether or not nicotine-related lncRNAs we identified are affected by the transcription factor NtMYC2, we performed quantitative PCR experiments in the roots of NtMYC2 mutation lines created through the CRISPR/Cas9 gene editing technology. The experimental results showed that eight lncRNAs were all significantly downregulated (Figure 6C), indicating that these lncRNAs were affected by the transcription factor NtMYC2. MSTRG.194637, MSTRG.207613, and MSTRG.244754 were the three genes with the most significant variation in expression. Seven lncRNAs are affected by MeJA treatment and transcription factor NtMYC2; these lncRNAs are MSTRG.181724, MSTRG.194637, MSTRG. 2004, MSTRG.207613, MSTRG.22251, and MSTRG .244754.

## Discussion

Although many lncRNAs have been identified in plants, including Arabidopsis, rice, maize, wheat, Populus trichocarpa, and cotton (Liu et al., 2012; Shuai et al., 2014; Wang et al., 2015; Deng et al., 2018; Huang et al., 2018; Wang et al., 2018), studies for modelling plant tobacco are few. Here, we systematically investigated the lncRNA in nicotine biosynthesis using a public tobacco RNA-seq dataset. Our analysis generated a relatively robust list of potential lncRNAs for tobacco. This set of lncRNAs will likely be helpful for functional genomics research or studying possible differences among tobacco varieties. We identified more than 30,000 putative lncRNA transcripts, including 24,084 lincRNAs, 350 incRNAs, and 5778 NATs. In previous works, Chen et al. found 7,423 non-redundant lncRNAs; among them, more than half of the sequences (∼54.38%) in their study are also included in our work.

Subgenome dominance is a phenomenon where one of the parental sub-genomes often retains significantly more genes and exhibits significantly higher expression, stronger purifying selection, and a lower DNA methylation level than those of the other sub-genomes in an allopolyploid genome (Cheng et al., 2018; Edger et al., 2018). Common tobacco is an allotetraploid plant with two subgenomes (T and S). The subgenome dominance of lincRNA identified in our study was asymmetrical compared with the coding genes and NATs. The lincRNAs showed a higher proportion of subgenome dominance; 68.71% of lincRNAs were expressed from the S subgenome. The NATs and protein-coding genes had a similar subgenome dominance level. The tobacco genome also showed an asymmetrical evolution with 55%–57% S origin and 43%–45% T origin (Sierro et al., 2014). lincRNA may play an essential role in this phenomenon.

The lncRNAs identified in our study showed a tissue-specific expression manner. Similar to finding in other species (Necsulea et al., 2014; Chen et al., 2020), the temporal expression profile of lncRNAs revealed that lncRNAs expressed in narrower time windows than protein-coding genes. It has been proposed that lncRNA expression signatures may accurately determine the developmental lineage and tissue origin because of their more tissue-specific expression pattern. High tissue-specific lncRNA expression supports the idea of their highly specialized, possible regulatory functions. It also allows for using lncRNAs as tissue type and state markers.

LncRNAs can regulate the expression of protein-coding genes in a cis or trans manner (Wang and Chang, 2011). In our study, many distant lncRNAs (>5 kb from the protein-coding gene) exhibited a strong correlation in expression with protein-coding genes, but whether these lncRNAs exert their function in trans or as enhancers or repressors needs to be further investigated. A small subset of lncRNAs and protein-coding genes (within 5 kb) strongly correlate at expression levels. The possible reason is their promoter regions have common regulatory elements and are cis-regulated by nearby genes. Further studies on the mechanisms underlying the coordinated transcription of lncRNA-protein-coding gene pairs should provide additional insights into the function of lncRNAs in plants.

Regarding the regulation of nicotine biosynthesis and other secondary metabolism processes in tobacco, the role of lncRNAs has rarely been demonstrated. Previous work mainly focused on constructing miRNA-circRNA-mRNA networks that regulate nicotine biosynthesis (Chen et al., 2019). Our results provide more evidence and detail of expression patterns for lncRNA candidates. Nicotine was synthesized in root tissue, and we proposed that lncRNA candidates related to nicotine biosynthesis may also act in root tissue. By WGCNA analysis, MEyellow was identified as a root-specifically expressed module (Figure 5B), containing many coding genes and lncRNA candidates involved in nicotine biosynthesis. A sub-network related to the nicotine pathway was constructed based on the well-known nicotine-related protein gene and further criteria (including PCC). We analyzed the differential expressed transcripts of topping, filtered our nicotine-related sub-network, and finally identified one NAT and seven lincRNA candidates (Table 3), which may be potentially highly relevant candidates for nicotine biosynthesis.

Topping induces tobacco to synthesize more nicotine. The mechanical damage caused by the topping activates the jasmonic acid biosynthesis pathway, and jasmonic acid activates the expression of transcription factors NtMYC2 associated with nicotine biosynthesis. If the lncRNAs identified by us are genuinely involved in nicotine biosynthesis, they should also be affected by jasmonic acid and NtMYC2. To confirm whether the nicotine-involved lncRNAs we identified are affected by NtMYC2, We performed quantitative PCR experiments in tobacco roots of MeJA treatment and NtMYC2 mutation lines. The experimental results showed that 7 of the lncRNAs were affected by MeJA and the transcription factor NtMYC2. These results suggested that these lncRNAs are genuinely involved in the pathway of nicotine biosynthesis directly or indirectly.

Our study provides a comprehensive landscape of lncRNAs and sheds light on the features and expression patterns of these lncRNAs in tobacco. Also, it complements the reference genome annotation of tobacco, which might further aid functional studies on different components’ regulation in plants. Meanwhile, we also offer potential lncRNA candidates involved in nicotine biosynthesis. Further investigations of their detailed function and regulation, including verification of interacting partners and regulators of lncRNAs, will elucidate their mechanism of action.

## Conclusion

In this study, we identified 30212 lncRNAs in tobacco and predicted the potential lncRNA involved in nicotine biosynthesis or transport by WGCNA combined with toping RNA-seq data. We found that lincRNA in tobacco evolved asymmetrically, with more expressed from the S sub-genome. Through quantitative PCR experiments, we further confirmed that seven nicotine-related lncRNAs are induced by MeJA and affected by transcription factor NtMYC2. These findings further deepen our understanding of the features and functions of lncRNAs and provide new candidates for regulating nicotine biosynthesis in tobacco.

## Data Availability

The datasets presented in this study can be found in online repositories. The names of the repository/repositories and accession number(s) can be found below: https://www.ncbi.nlm.nih.gov/bioproject, PRJNA870299.
